# Online media reporting of suicidal behaviour in Ghana: Analysis of adherence to the WHO guidelines

**DOI:** 10.1177/0020764020919787

**Published:** 2020-05-16

**Authors:** Emmanuel Nii-Boye Quarshie, Johnny Andoh-Arthur, Kwaku Oppong Asante, Winifred Asare-Doku

**Affiliations:** 1 School of Psychology, University of Leeds, Leeds, UK; 2 Centre for Suicide and Violence Research (CSVR), Accra, Ghana; 3 Department of Psychology, University of Ghana, Accra, Ghana; 4 Department of Psychology, University of the Free State, Bloemfontein, South Africa; 5 School of Medicine and Public Health, The University of Newcastle, Callaghan, NSW, Australia

**Keywords:** Attempted suicide, completed suicide, Ghana, online media, sub-Saharan Africa, suicide reporting

## Abstract

**Background:**

Irresponsible media reporting of suicide is a potential risk for copycat suicide. There is a paucity of studies from sub-Saharan Africa on the quality of media reporting of suicide.

**Objectives:**

We assessed the compliance of Ghanaian online media outlets with the World Health Organization (WHO) guidelines for media reporting of suicide.

**Methods:**

We searched 10 local media outlets with strong online presence in Ghana, to identify suicide-related news reports from 2000 through 2019. We applied summative content analysis and chi-square $\left(\chi^{2}\right)$ test to the data.

**Results:**

We included 288 news reports, of which 261 (90.6%) were completed suicides, 7 (2.4%) were attempted suicides and 20 (6.9%) were homicide suicides. Most of the news reports failed to comply with the WHO guidelines: 92.7% mentioned the specific method of the suicide act, 82.6% included ‘suicide’ in the headline and 55.6% included photos of the victims. The $\chi^{2}$ tests indicated that privately owned media outlets were more likely than publicly owned to post a photo of the victim, $\chi^{2}(1)$ = 17.37, *p* < .001, and report the incident location in the headline, $\chi^{2}(1)$ = 15.00, *p* < .001. However, generally, there were no statistically significant relationships between the quality of reporting and media outlet ownership. Each of the 288 reports failed to mention any of the potentially helpful features recommended by the WHO guidelines.

**Conclusion:**

Regardless of the ownership of the media outlet (whether private or publicly owned), mostly, the online reportage of suicidal behaviour in Ghana deviates sharply from the international recommended best practice by the WHO.

## Introduction

Suicide is newsworthy (Machlin et al., 2013; Sullivan, 2007); however, irresponsible media reporting of the phenomenon has been implicated as a risk factor for death by suicide, particularly, in vulnerable groups within the population (Niederkrotenthaler et al., 2009, 2010; Sinyor, Schaffer, Nishikawa, et al., 2018; Sisask & Värnik, 2012; World Health Organization [WHO], 2014). Studies have consistently identified that persons experiencing suicidal crisis could become aware of suicide methods, which they might have not thought about previously, through exposure to irresponsible media reporting of suicide, thereby increasing the chances of copying the suicidal behaviour (Cheng et al., 2017, 2018; Niederkrotenthaler et al., 2012; Nutt et al., 2015; Pirkis, Burgess, et al., 2006; Tsai, 2010; WHO & International Association for Suicide Prevention [IASP], 2017). This form of social learning has been found mainly among students and young people (Cheng et al., 2018; Gould et al., 2014; O’Connor et al., 2014; Zalsman et al., 2016).

The suicide reporting guidelines of the WHO provide standards for responsible media reporting of suicide around the world (WHO, 2000; WHO & IASP, 2017), but several countries have developed context-specific standard guidelines for professional media practitioners (Mishara & Dargis, 2019; Pirkis, Blood, et al., 2006; Samaritans, 2013; Sinyor, Schaffer, Heisel, et al., 2018; Utterson et al., 2017). The WHO reporting guidelines outline certain potentially helpful features, which may help prevent suicide, and potentially harmful characteristics, which may provoke suicide, thereby providing a source of education on best practice for media practitioners (WHO, 2000; WHO & IASP, 2017). Evidence from high-income countries suggests that guidelines on media reporting of suicide have been useful, although reliable evaluative evidence on the impact of the guidelines on professional practice of media practitioners and on suicide and suicidal behaviours remains limited (Bolzern et al., 2019; Marzano et al., 2018; Niederkrotenthaler et al., 2010; Pirkis, Blood, et al., 2006; Pirkis et al., 2017; Stack, 2020; Utterson et al., 2017; Williams & Witte, 2018).

In most low- and middle-income countries (LAMICs), the WHO reporting guidelines remain the main reference document guiding media practitioners in reporting suicide. Similar to research findings from many high-income countries (Bolzern et al., 2019; Marzano et al., 2018; McTernan et al., 2018; Stack, 2020; Utterson et al., 2017), recent evidence shows that adherence to the WHO’s recommendations and compliance with standard reporting guidelines are still lacking among journalists and media outlets in LAMICs (Arafat et al., 2019; Armstrong et al., 2018; Chandra et al., 2014; Chang & Freedman, 2018; Cheng et al., 2017; Chiang et al., 2016; Chu et al., 2018; Chun et al., 2018; Yang et al., 2013). For example, quality assessment of media reporting of suicide in India, within the lens of the WHO reporting guidelines, shows that reporting of potentially harmful features (e.g. details of suicide method) is common, while recommended helpful reporting practices are scarce – for example, providing contact details of available and accessible suicide support services (Armstrong et al., 2018; Chandra et al., 2014). In Bangladesh, Bhutan, China, India, Indonesia, Korea, Sri Lanka and Taiwan, both online and newspaper reporting of suicide have been found to fail in adhering to the WHO recommended reporting guidelines (Arafat et al., 2019; Chandra et al., 2014; Chang & Freedman, 2018; Chu et al., 2018; Chun et al., 2018; Nisa et al., 2020; Sørensen et al., 2019; Zangmo & Zangmo, 2019).

Recent evidence from Bhutan, India, Indonesia and Sri Lanka indicates that although some media reports show some level of adherence to recommended reporting guidelines, the majority of the available media reports are non-compliant (Chandra et al., 2014; Nisa et al., 2020; Sørensen et al., 2019; Zangmo & Zangmo, 2019). Frequently, they reported the method of self-harm and suicide in the headline, included identifiable information (e.g. picture) of the victim, used sensational and insensitive language and attributed a single-factor cause to the self-harm or suicide, without providing any information on help-seeking and available support services.

The majority of news reports on suicide in Korea mentioned the method used and provided specific details about the location where the suicidal act took place, and almost half of the reports that were analysed mentioned the contents of suicide notes, while less than 3% of the news reports provided information about helplines, research evidence about suicide and suicide prevention (Chun et al., 2018). Similarly, in Bangladesh, most of the news reports mentioned the names of victims, victims’ occupations and provided details of the suicide method; others reported life events and made monocausal attributions of suicide, the term ‘suicide’ appeared in the headlines of the majority of the reports, some of the reports posted pictures of victims, but no news report provided any potentially helpful recommended information – for example, expert opinion or information about suicide prevention (Arafat et al., 2019, 2020a, 2020b; Arafat, Khan, et al., 2020). Besides these breaches of the recommended reporting practices being associated with increased chances of copycat suicide and imitative suicidal behaviours among vulnerable groups (Niederkrotenthaler et al., 2010, 2012; Pirkis, Blood, et al., 2006), loved ones and families bereaved by suicide and the general public have reported being upset by the media providing details of the death, posting photos of the deceased and the general careless and insensitive nature of media reporting of suicide (Chang & Freedman, 2018; Chapple et al., 2013; Gregory et al., 2020).

Furthermore, the WHO and the IASP caution against the use of the ‘c-word’ (‘committing’, ‘commits’ or ‘committed’ suicide), as the phrase ‘committed suicide’ connotes criminality and a moral sin and reinforces stigma (WHO & IASP, 2017). In place of c-words, neutral terms such as ‘ended/took her or his life’ are suggested (Beaton et al., 2013). Despite this caution, there are reports that the use of the c-word with suicide is still common, particularly, in media reports of suicide (Beaton et al., 2013; Nielsen et al., 2016; WHO & IASP, 2017).

There is a paucity of studies from sub-Saharan African countries on the quality of media reporting of suicide, while no published study from Ghana has specifically examined the quality and compliance of media reporting of suicidal behaviours with any known standard reporting guidelines (Akotia et al., 2019; Quarshie et al., 2015). In the present study, we assessed the compliance of Ghanaian online media outlets with the WHO guidelines for media reporting of suicide, by performing a summative content analysis of the frequency and patterns of both breaches and recommended contents of news reports on suicide, attempted suicide and homicide suicide.

## Methods

### Design and data source

We reviewed the contents of news reports on suicide and suicidal behaviours in Ghana from 2000 through 2019. Two authors (E.N.-B.Q. and W.A.-D.) performed keyword searches in January 2020 of the portals of 10 local media outlets with strong online presence in Ghana. Three of the media outlets were publicly owned (*Daily Graphic, Ghana News Agency* and *Ghanaian Times*), while seven were privately owned (Adom FM, Citi Newsroom, Daily Guide Africa, GhanaWeb, MyJoyOnline, Peace FM and Starr FM). Previous studies have found the news portals of these media houses as having wider readership and listenership among Ghanaians (Quarshie et al., 2015, 2018). We retrieved eligible news reports about completed suicide, attempted suicide and homicide suicide; we excluded news reports about euthanasia, and suicide bombing, as were also editorials and opinion pieces. English is the official language of Ghana and the language for all written media contents in the country. Across the review period, we identified a total of 513 potentially eligible news reports. We independently assessed the eligibility of the total hits and identified 288 news reports included in the final analysis of this study.

### Data extraction, coding and analysis

Consistent with the WHO guidelines for media reporting of suicide (WHO, 2000; WHO & IASP, 2017), we followed the summative content analysis procedure recommended by Hsieh and Shannon (2005) to identify potentially harmful characteristics (breaches) and potentially helpful characteristics (recommended features) in each of the included 288 news reports. Potentially harmful characteristics identified in the news reports were related to breaches of the WHO reporting guidelines, for example, mention of the term ‘suicide’ in the headline, provision of detailed account of suicide method used, giving monocausal explanation for suicidality, romanticising or glamorising suicide, mention of the age, name, school/workplace of victims, posting a photo of the victim or a photo symbolic of self-harm/suicide (e.g. hangman’s noose) and inclusion of additional problematic online features (e.g. links to other articles/websites about suicide, reader-generated comment threads).

We also examined each news story for potentially helpful characteristics related to providing mental health literacy (e.g. dispelling common myths about suicide or providing public education about the facts of suicide), drawing on health experts’ views, research and data to inform public and promoting help-seeking behaviour (e.g. raising awareness of crisis support or prevention services available). In accordance with the WHO reporting guidelines, we included each of the identified breaches and recommended features as the variables of the analysis, and coded absent (0) or present (1).

Using the Statistical Package for Social Sciences (SPSS, Version 26.0, for Windows), we analysed the data for the frequencies and patterns of both breaches and recommended contents of the included news reports. We applied chi-square $\left(\chi^{2}\right)$ test to compare the compliance of suicide reporting between public and privately owned online media; we used Fisher’s exact test where a cell expected frequency was <5 (Fleiss et al., 2003). The conventional statistical threshold of *p* < .05 was used to determine statistically significant results.

### Ethics

The protocol guiding this study was not submitted to an Institutional Review Board for ethical approval, as the study did not include the recruitment and involvement of human participants. However, in developing this article, we have preserved the ethical position of this study by excluding identifying information of media outlets, and suicidal persons and their families mentioned in the included news reports (e.g. names, address, place of work or school).

## Results

A total of 288 news reports drawn from public (*n* = 62 (21.5%)) and privately owned (*n* = 226 (78.5%)) online media outlets met the inclusion criteria for this study. Of the 288 news reports, 261 (90.6%) were completed suicides, 7 (2.4%) were attempted suicides and 20 (6.9%) were homicide suicides. Of the 261 completed suicides, 13 (5%) were identified as celebrity suicides.

Table 1 presents the quality assessment of reporting compliance with WHO guidelines for media reporting of suicide. Generally, most of the included news reports failed to comply with the WHO guidelines: 82.6% included the term ‘suicide’ in the headline, as was a ‘c-word’ included in the headlines of 79.9%. While 92.7% of the news reports mentioned the specific method of the suicide act, 66.3% provided detailed descriptions of the methods used for the act. Also, 92.7% of the news reports mentioned the specific place or location where the suicidal act took place. Furthermore, 83.7% reported the names of the victims, 55.6% included photos of the victims and 50.3% provided reader-generated comment threads.

**Table 1.** Quality assessment of reporting compliance with WHO suicide reporting guidelines.

Potentially harmful breaches	Absent			Present			$\chi^{2}$ test	*p* value
	Total	Public media	Private media	Total	Public media	Private media		
	*n* (%)	*n* (%)	*n* (%)	*n* (%)	*n* (%)	*n* (%)		
Headlines								
‘Suicide’ in the headline	50 (17.4)	8 (16.0)	42 (84.0)	238 (82.6)	54 (22.7)	184 (77.3)	1.094	.296
Suicide method in the headline	187 (64.9)	57 (30.5)	130 (69.5)	101 (35.1)	5 (5.0)	96 (95.0)	25.304	.000
Life event(s) in the headline	216 (75.0)	50 (23.1)	166 (76.9)	72 (25.0)	12 (16.7)	60 (83.3)	1.343	.247
‘C-word’ in the headline^a^	58 (20.1)	8 (13.8)	50 (86.2)	230 (79.9)	54 (23.5)	176 (76.5)	2.572	.109
Incident location	151 (52.4)	46 (30.5)	105 (69.5)	137 (47.6)	16 (11.7)	121 (88.3)	15.004	.000
Suicidal act (as presented in the body of story)								
Suicide method reported	21 (7.3)	3 (14.3)	18 (85.7)	267 (92.7)	59 (22.1)	208 (77.9)	0.701	.437
Detailed account of method	97 (33.7)	14 (14.4)	83 (85.6)	191 (66.3)	48 (25.1)	143 (74.9)	4.358	.037
Place or public site named as location of a suicide death/attempt	21 (7.3)	3 (14.3)	18 (85.7)	267 (92.7)	59 (22.1)	208 (77.9)	0.701	.437
Time of the act (e.g. morning, afternoon, night)	142 (49.3)	33 (23.2)	109 (76.8)	146 (50.7)	29 (19.9)	117 (80.1)	0.486	.486
Suicidal behaviour described as ‘successful’ or ‘unsuccessful’	232 (80.6)	62 (26.7)	170 (73.3)	56 (19.4)	0	56 (100)	19.071	.000
Causes of suicidality								
Monocausal explanation for suicidality	180 (62.5)	43 (23.9)	137 (76.1)	108 (37.5)	19 (17.6)	89 (82.4)	1.584	.208
Narrative brushes over the complex realities of suicide	232 (80.6)	52 (22.4)	180 (77.6)	56 (19.4)	10 (17.9)	46 (82.1)	0.554	.457
Contents/details from suicide note reported	267 (92.7)	58 (21.7)	209 (78.3)	21 (7.3)	4 (19.0)	17 (81.0)	0.082	.774
Consideration for bereaved persons								
Romanticising or glamorising suicide or over-emphasising family/community expressions of grief	217 (75.3)	48 (22.1)	169 (77.9)	71 (24.7)	14 (19.7)	57 (80.3)	0.183	.669
Interview with bereaved persons (comments from survivors or bereaved)	130 (45.1)	33 (25.4)	97 (74.6)	158 (54.9)	29 (18.4)	129 (81.6)	2.087	.149
Disclosure of victim’s identity								
Name of suicidal person mentioned	47 (16.3)	9 (19.1)	38 (80.9)	241 (83.7)	53 (22.0)	188 (78.0)	0.188	.661
Age of suicidal person mentioned	53 (18.4)	15 (28.3)	38 (71.7)	235 (81.6)	47 (20.0)	188 (80.0)	1.764	.184
Occupation or student status mentioned	88 (30.6)	12 (13.6)	76 (86.4)	200 (69.4)	50 (25.0)	150 (75.0)	4.671	.031
Name of school or place of work mentioned	148 (51.4)	26 (17.6)	122 (82.4)	140 (48.6)	36 (25.7)	104 (74.3)	2.826	.093
Photo of victim	128 (44.4)	42 (32.8)	86 (67.2)	160 (55.6)	20 (12.5)	140 (87.5)	17.368	.000
Celebrity status								
Victim identified as a celebrity	275 (95.5)	60 (21.8)	215 (78.2)	13 (4.5)	2 (15.4)	11 (84.6)	0.303	.741
Victim related to or has link with a celebrity	283 (98.3)	61 (21.6)	222 (78.4)	5 (1.7)	1 (20.0)	4 (80.0)	0.007	1.000
Additional potentially harmful features								
Link(s) to other articles/websites about suicide	273 (94.8)	62 (22.7)	211 (77.3)	15 (5.2)	0	15 (100)	4.326	0.47
Reader-generated comment threads	143 (49.7)	25 (17.5)	118 (82.5)	145 (50.3)	37 (25.5)	108 (74.5)	2.751	.097
Suggests or encourages a suicide cluster	268 (93.1)	58 (21.6)	210 (78.4)	20 (6.9)	4 (20.0)	16 (80.0)	0.030	1.000
Potentially helpful features (recommended features)								
Mental health literacy								
Dispels the myths about suicide or educate the public about the facts of suicide	288 (100)	62 (21.5)	226 (78.5)	–	–	–	–	–
Draws on health experts, research and data to inform public	288 (100)	62 (21.5)	226 (78.5)	–	–	–	–	–
Promoting help-seeking behaviour								
Mentions a suicide prevention programme/support service	288 (100)	62 (21.5)	226 (78.5)	–	–	–	–	–
Provides contact details for a suicide support service	288 (100)	62 (21.5)	226 (78.5)	–	–	–	–	–

Note: WHO: World Health Organization; $\chi^{2}$: chi-square.

a The c-word criterion refers to the mention of ‘commit’ suicide, ‘committed’ suicide or ‘committing’ suicide in the headline of the news report.

Surprisingly, each of the 288 news reports analysed failed to mention any of the potentially helpful features recommended by the WHO reporting guidelines (e.g. providing public education about the facts of suicide, mentioning a suicide prevention programme or support service, or providing contact details for a suicide support service). The $\chi^{2}$ tests indicated that privately owned online media outlets were more likely than those publicly owned to report the incident location in the news headline, $\chi^{2}(1)$ = 15.00, *p* < .001; include a photo of the victim, $\chi^{2}(1)$ = 17.37, *p* < .001; and provide a detailed account of the method used for the suicidal act, $\chi^{2}(1)$ = 4.36, *p* < .05. However, generally, across the range of variables analysed, the $\chi^{2}$ results did not show statistically significant relationships between the quality of reporting and media outlet ownership.

## Discussion

While it is relevant to compare the evidence of the present study to findings of the large number of previous international studies – mainly, from high-income countries – it may be more meaningful to discuss the evidence of our study in the light of the available evidence from LAMICs and the context of Ghana. The present study shows that, generally, the media reporting of suicidal behaviour in Ghana does not comply with the WHO reporting guidelines. This evidence supports recent findings from other LAMICs in Asia, including Bangladesh, Bhutan, India, Indonesia, Sri Lanka and Taiwan (Arafat et al., 2019; Chiang et al., 2016; Nisa et al., 2020; Sørensen et al., 2019; Zangmo & Zangmo, 2019). The evidence also supports a previous anecdotal observation that media coverage of suicide in Ghana is sensational, overly simplistic and explicit (Quarshie et al., 2015). The predominant inclusion of the c-word and the term ‘suicide’ in the headlines as evident in the present study is particularly troubling, even though not entirely surprising. Recent evidence indicates that news editors in Ghana consider ‘suicide’ as a catchy term which attracts reader attention (Akotia et al., 2019), while some emerging researchers of suicide in the country, without background training in suicidology, have been found to use the c-word in scientific publications (e.g., Abdulai, 2020; Acheampong & Aziato, 2018; Der et al., 2016). As pertaining in many LAMICs (Mishara & Weisstub, 2016), suicide is highly stigmatised and morally tabooed, and attempted suicide is criminalised in Ghana (Act 29 of Ghana, 1960; Adinkrah, 2013; Osafo et al., 2015). Thus, the inclusion of the c-word and ‘suicide’ in the headlines of news reports of suicidal behaviour has implications for potential inadvertent deepening of the criminality and moral condemnation of victims and survivors of (attempted) suicide and their families (Beaton et al., 2013; Nielsen et al., 2016).

As found in other LAMICs (Chandra et al., 2014), generally, the present study found no statistically significant relationships between the quality of reporting and media outlet ownership – a plausible indication that the extent of non-compliance with recommended best practice transcends the ownership of media outlets. It is possible that today’s fiercely competitive media terrain and the extreme commercial motive of media outlets could be responsible for this sensationalistic media practice – every media house, public or privately owned, wants to be the first to break the news on suicide (Akotia et al., 2019; Yang et al., 2013). Relatedly, the disclosure of victim’s identity (mention of names, school, place of work or posting of photos) remains a critical ethical issue; some researchers have interpreted this practice to mean journalists and media outlets exploiting the suicide event for commercial gains, while remaining uncompassionate to the bereaved family (Akotia et al., 2019; Harshe et al., 2016).

Furthermore, the mention and detailed description of the method and location of suicide have been identified as cause for concern. Narrative and systematic reviews have identified the existence of the Werther effects in vulnerable groups (Blood et al., 2007; Cheng et al., 2017; Niederkrotenthaler et al., 2010, 2012; Pirkis et al., 2009; Sisask & Värnik, 2012; Stack, 2003), while adolescents with histories of self-harm have attributed their knowledge of self-harm methods to media reporting and portrayal of suicide (O’Connor et al., 2014). In online media reporting of suicide, the additional inclusion of links to other articles/websites about suicide and reader-generated comment threads could be problematic (Utterson et al., 2017). Evidence suggests that the online discussion forums are associated with suicidal behaviours in young people (Dunlop et al., 2011).

Finally, the evidence of the present study that each of the 288 media reports analysed failed to mention any of the potentially helpful features recommended by the WHO guidelines is worthy of some comments. This finding is consistent with recent evidence from Bangladesh, India and Indonesia, where no news report analysed provided any potentially helpful information recommended by the WHO guidelines (Arafat et al., 2019, 2020a, 2020b; Chandra et al., 2014; Nisa et al., 2020; Sørensen et al., 2019). Perhaps in Ghana, this troubling evidence highlights the need for the country’s Ministry of Health to consider launching stakeholder training programmes and sustained awareness creation among media practitioners on the importance of adhering to recommended standard guidelines for media reporting of suicidal behaviour.

### Limitations and recommendations

The non-inclusion of media outlets with lesser online presence in Ghana might have limited the evidence of this study, regarding the extent and pattern of compliance with or breaches of the WHO guidelines. The present study fails to assess the effects of non-compliant media reporting of suicide on the actual rates of suicide and suicidal behaviours in Ghana. Future studies may consider the use of ecological approaches and longitudinal designs to examine the copycat effects of non-compliant media reporting of suicide in Ghana.

Responsible media reporting of suicide is now a key area of focus for suicide prevention among vulnerable groups (Oexle et al., 2019; WHO, 2014; Zalsman et al., 2016). Thus, the WHO and leading researchers in the area of media and suicide prevention have recommended the development of media reporting guidelines by countries around the world (Chu et al., 2018; Mishara & Dargis, 2019; Pirkis, Blood, et al., 2006; WHO, 2014). The key evidence of the present study underscores the urgent need for mental health professionals and key media role players in Ghana (the Ghana Mental Health Authority, Ghana Psychological Council, Ghana Media Commission, National Communication Authority, the Ghana Journalists Association, training institutions of media practitioners and the Centre for Suicide and Violence Research) to collaborate towards the formulation, adoption and implementation of guidelines for responsible (online) media reporting of suicide in the country.

## Conclusion

The evidence of the present study shows that, in Ghana, online media reporting of suicidal behaviour deviates sharply from the international recommended best practice by the WHO; these reports use sensationalistic language and crude practices that are insensitive to the needs of loved ones and families bereaved by suicide and may have the inherent tendency of triggering copycat suicide in vulnerable groups.
